# A Treatise on Diseases of the Nose and Throat

**Published:** 1900-12

**Authors:** 


### BOOK REVIEWS, PAMPHLETS, EXCHANGES.

### BOOK REVIEWS.

[Contributions solicited for review.]

A Treatise on Diseases of the Nose and Throat. By E. L. Shurly, M.D., Professor of Laryngology and Clinical Medicine in Detroit College of Medicine. Published by D. Appleton & Co., N. Y.

The American medical profession can certainly boast of voluminous writers of text-books from among its members. The department of laryngology and rhinology has most assuredly maintained its share of contributions. A number of excellent text-books have been published on these branches during the last two or three years, and it is almost impossible to imagine how any author could have the temerity to swell this number by an additional work. It may be that the publishers see a demand for new books, and it may be that the growth in the number of the medical profession demands an increased number of text-books, but as yet the writer of this review fails to comprehend such a necessity. It is rather amusing to note the preface of every new medical work issued, in that we always find such beginning with an apology and trying to impress upon its readers the real cause for such a publication. These remarks are made not especially in reference to the above treatise, but have reference to nearly all the modern text-books. This work by Dr. Shurly is one of great excellence. The subjects are all treated of in a most masterful way, and we are free to admit that it is one of the best modern works on the nose and throat that it has been our privilege to peruse. The illustrations are most excellent and well adapted to illustrate the subject. Especially to be mentioned is the chapter on intubation, which is exceedingly clear in detail, and the accompanying cut and photographs the best we have ever seen. The publishers have done their work well, and altogether we congratulate Dr. Shurly on the success of this volume. Roy.

